# Microbial Shelf-Life, Starch Physicochemical Properties, and In Vitro Digestibility of Pigeon Pea Milk Altered by High Pressure Processing

**DOI:** 10.3390/molecules25112516

**Published:** 2020-05-28

**Authors:** Yun-Ting Hsiao, Chung-Yi Wang

**Affiliations:** Department of Biotechnology, National Formosa University, Yunlin 632, Taiwan; cdd828@nfu.edu.tw

**Keywords:** glycemic index, high pressure processing, pigeon pea milk, resistant starch

## Abstract

This study examined the effects of high-pressure processing (HPP) on microbial shelf-life, starch contents, and starch gelatinization characteristics of pigeon pea milk. HPP at 200 MPa/240 s, 400 MPa/210 s, and 600 MPa/150 s reduced the count of *Escherichia coli* O157:H7 in pigeon pea milk by more than 5 log CFU/mL. During the subsequent 21-day refrigerated storage period, the same level of microbial safety was achieved in both HPP-treated and high-temperature short-time (HTST)-pasteurized pigeon pea milk. Differential scanning calorimetry and scanning electron microscope revealed that HPP at 600 MPa and HTST caused a higher degree of gelatinization in pigeon pea milk, with enthalpy of gelatinization (∆H) being undetectable for both treatments. In contrast, HPP at 400 MPa led to an increase in the onset temperature, peak temperature, and conclusion temperature, and a decrease in ∆H, with gelatinization percentages only reaching 18.4%. Results of an in vitro digestibility experiment indicate that maximum resistant starch and slowly digestible starch contents as well as a decreased glycemic index were achieved with HPP at 400 MPa. These results demonstrate that HPP not only prolongs the shelf-life of pigeon pea milk but also alters the structural characteristics of starches and enhances the nutritional value.

## 1. Introduction

The pigeon pea (*Cajanus cajan* (L.) Millsp.), which belongs to the genus Cajanus of the family Fabaceae, is a perennial shrub commonly found in the villages of indigenous tribes in the mountainous areas in Asia. It is also the only woody plant species that bears edible legumes. With its high nutritional value, pigeon pea has been regarded as having heat-clearing, detoxification, diuretic, edema alleviation, spleen and stomach invigoration, qi replenishment, bleeding control, and diarrhea relief effects in Traditional Chinese Medicine (TCM) practices. Previous research has indicated that flavonoids are the main health-promoting components in pigeon pea. A total of 27 flavonoids have been isolated from pigeon pea, with cajaninstilbene acid, orientin, pinostrobin, and vitexin as being the four most abundantly present flavonoid components. It has also been reported that the flavonoid content of pigeon pea has antioxidant capacities such as free radical scavenging ability, reducing ability, and inhibition of lipid peroxidation [1,2]. In a study by Patel et al. [3], it was found that pinostrobin and cajanus lactone isolated from pigeon pea leaves had anti-inflammatory effects, and were capable of reducing TNF-α and IL-1β levels in lipopolysaccharide (LPS)-stimulated RAW 264.7 and J774A.1 cells. The results of another study also showed that cajanuslactone and sesquiterpenes isolated from pigeon pea leaves possessed good antibacterial activity against pathogenic bacteria such as *Staphylococcus* aureus, *Bacillus subtilis*, and Propionibacterium [4]. Lee et al. [5] found that pigeon pea fermented using *Bacillus subtilis* (natto) produced natto kinase and exhibited an increase in antioxidant ability, angiotensin-converting enzyme inhibitory activity, and antihypertensive effects, which demonstrated the application potential of *Bacillus*-fermented pigeon pea in the development of health-promoting foods. Several animal model experiments have shown that the intake of pigeon pea in experimental hamsters provided significant health-promoting effects. In a study by Ariviani et al. [6], the potential use of a pigeon pea beverage as an anti-diabetic functional drink was investigated through the administration of the pigeon pea beverage to rats for three months. Results indicated that the pigeon pea beverage showed hypoglycemic and hypocholesterolemic activities, which improved the antioxidant status of diabetic-hypercholesterolemia rats, with the plasma glucose, total cholesterol, and plasma MDA (malondialdehyde) levels of diabetic-hypercholesterolemia rats decreased by 33.86%, 19.78%, and 37.16%, respectively. Dai et al. [7] found that the administration of pigeon pea to high-fat diet fed hamsters led to a significant decrease in LDL cholesterol levels; with the effect increases with higher doses of pigeon pea. The underlying regulatory mechanism may be attributed to the promotion of hypolipidemic activity and bile acid synthesis through the regulation of LDL receptor and cytochrome P450 7A1 (CYP7A1) expression. In a similar study, different doses of pigeon pea extracts were continuously administered to mice with diet-induced hypercholesterolemia for four weeks. Results indicated that serum LDL cholesterol was significantly reduced, whereas the mRNA expressions of HMG-CoA reductase, CYP7A1, and LDL-receptor were markedly enhanced, which increases the efficiency of lipid and cholesterol metabolism in the mice, and led to the alleviation of hyperlipidemia [8].

High pressure processing (HPP) is a non-thermal pasteurization technique which involves the application of an ultra-high pressure (100 MPa or more) at room temperature to food products sealed in flexible packaging to achieve the inactivation of pathogenic microbes. Compared to traditional thermal processing methods, HPP is carried out at room temperature, thereby eliminating the need for energy consuming-processes such as heating and cooling. Furthermore, food products are prepackaged prior to HPP whereby direct contact with the processing equipment is completely avoided, therefore minimizes the risk of secondary contamination [9]. Hence, HPP treatment maintains food safety and prolongs food products shelf-life in cold chain storage and circulation while preserving the original flavors and nutritional value of the products. HPP is now widely applied in the production of meat, dairy, aquatic, fruit, vegetable products as well as beverages; and the myriad of collaborative research reports has shown that HPP has significant inhibitory effects on common foodborne pathogens such as *Staphylococcus aureus*, *E. coli*, *Salmonella*, *Vibrio parahaemolyticus*, and *Listeria monocytogenes* [10]. In the production of ready-to-drink beverages, many manufacturers have adopted HPP to prolong shelf-life and to minimize the effects of high temperature on the natural flavors of food products. A study by Smith et al. [11] showed that a four-fold increase in the shelf-life of soymilk could be achieved with high pressure pasteurization. Furthermore, through sensory evaluation, it was found that HPP-treated soymilk smoothies had no significant differences in color, aroma, and antioxidant attributes compared to freshly made soymilk smoothies [12,13].

Due to its drought tolerant characteristics, pigeon pea is considered one of the key food crops in the arid regions of many developing countries in Asia, Africa, and Latin America. Pigeon pea has also shown high potential used for the production of natto, noodle, and bakery products [14,15,16]. In the present study, pigeon pea milk was first prepared from pigeon peas and subsequently subjected to various treatments to evaluate the effects of HPP on microbial safety, physicochemical properties of starches such as the degree of starch gelatinization, and resistant starch content, and in vitro digestibility of pigeon pea milk. Changes in the morphology of starch granules in HPP-treated pigeon pea milk were also observed using an electron microscope to elucidate the mechanisms by which HPP alters the starch textures of pigeon pea.

## 2. Results

### 2.1. HPP Conditions

**Figure 1:**
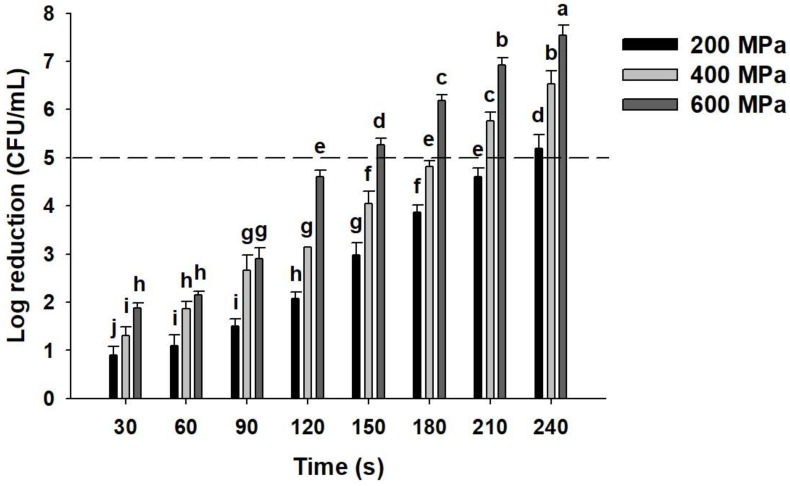
Effects of high-pressure processing on *E. coli* O157H7 in pigeon pea milk. Data are means of three determinations ± standard deviation (*n* = 3). Bars with different superscript letters are significantly different (*p* < 0.05).

From Figure 1, it can be seen that an increase in the treatment pressure and duration of HPP led to the reduction in viable count. The application of HPP at 200, 400, and 600 MPa for 150 s led to the reductions in *E. coli* O157:H7 viable count by 2.98, 4.05, and 5.27 log CFU/mL, respectively. When treatment duration was extended to 210 s, HPP at 200 and 400 MPa resulted in the reductions of 4.60 and 5.76 log CFU/mL, respectively. A treatment duration of 240 s was required for HPP at 200 MPa to achieve a 5.2 log reduction in *E. coli* O157:H7, which is no significant different with the HPP condition of 600 MPa for 150 s. The result shows that HPP at 200 MPa/240 s, 400 MPa/210 s, and 600 MPa/150 s reduced the count of *E. coli O157:H7* in pigeon pea milk by more than 5 log CFU/mL. According to the recommendations provided by the United States Department of Agriculture (USDA), appropriate indicator bacteria for HPP-pasteurized products should be selected for the investigation of pressure resistance characteristics as well as the analysis of bactericidal kinetic parameters. Processing conditions that enable at least a 5-log reduction of the indicator bacteria is regarded as the minimum criteria of food hygiene and safety requirements. *E. coli* O157:H7, a common foodborne pathogen, is frequently associated with food poisoning. Due to its relatively high pressure resistance compared to other pathogenic bacteria, many manufacturers of HPP-treated food products have selected *E. coli* O157:H7 as the indicator bacterial strain for the formulation of appropriate HPP conditions (USDA, 2012). In the present study, HPP conditions that enabled the achievement of 5-log reductions in *E. coli* O157:H7 were adopted during subsequent experimentation for the investigation of pigeon pea starch characteristics.

### 2.2. Microbial Shelf-Life

Table 1 shows the changes in the bacterial counts of the various pigeon pea milk samples during refrigerated storage at 4 °C, over a 28-day period. For fresh pigeon pea milk (control group), the initial total aerobic bacteria (TAB), psychrotrophic and *E. coli*/coliform counts were 1.65, 1.34, and 0.35 log CFU/mL, respectively, which increased to 5.42, 3.82, and 1.77 log CFU/mL after 28 days of storage. When pigeon pea milk samples were separately subjected to HPP at 200 MPa for 240 s, 500 MPa for 210 s, and 600 MPa for 150 s, the TAB counts of the samples were immediately reduced to <1.0 log CFU/mL, with the growth of residual bacteria only detected in the HPP-200 and HPP-400 groups from Day 7 onwards. By Day 28, the TAB counts of the HPP-200, HPP-400, and HPP-600 groups had increased to 1.12, 0.78, and 0.92 log CFU/mL, respectively. HTST pasteurization completely inhibited TAB growth within the first 14 days of storage, with the growth of residual bacteria only detected from Day 21 onwards, and the TAB count remaining below 1.0 log CFU/mL up to Day 28. Both HPP and HTST treatments managed to reduce the psychrotrophic count beyond the detection limit. Throughout the 28-day refrigerated storage period, the psychrotrophic count of HPP-treated pigeon pea milk did not increase significantly; with the growth of psychrotrophs only detected from Day 14 onwards in the HPP-200 and HPP-400 groups. In contrast, the growth of psychrotrophs was only observed on Day 28 in the HTST group, with the psychrotrophic count being <1.0 log CFU/mL. The *E. coli*/coliform counts of the various groups during the storage period followed a trend similar to that of the psychrotrophic counts. During the first 21 days of storage, the HPP-400 and HPP-600 groups achieved the same pasteurization effects as the HTST group, whereas the HPP-200 group had an *E. coli*/coliform count of 0.33 log CFU/mL on Day 21. However, growth was detected in the residual TAB, psychrotrophs and *E. coli*/coliforms of all HPP treatment groups from Day 28 onwards. Although all bacterial counts were below 1.0 log CFU/mL, these results indicate that the recommended shelf-life of HPP-treated pigeon pea milk should not exceed 21 days to maintain the minimum microbial safety.

### 2.3. Starch Gelatinization Characteristics

**Figure 2:**
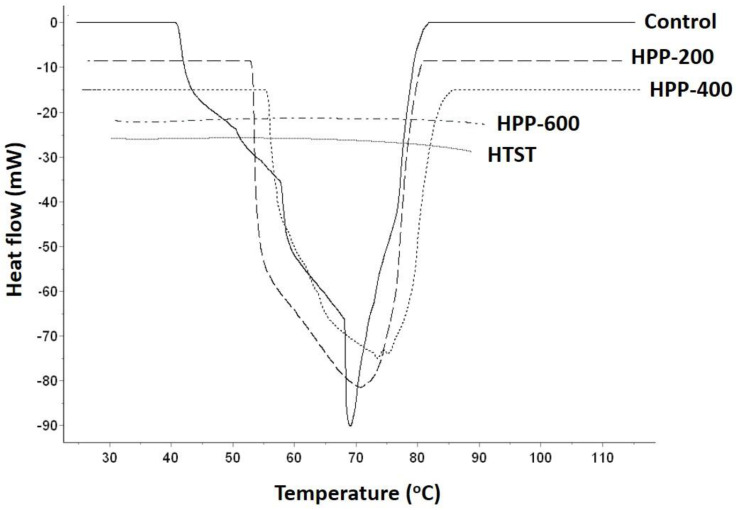
DSC thermograms of control, HTST, and HPP treated pigeon pea milk starch granules.

Figure 2 shows the DSC thermograms of starches obtained from untreated, HPP-treated, and HTST-treated pigeon pea milk samples, and Table 2 shows the gelatinization onset (To), peak (Tp), and conclusion temperatures (Tc); gelatinization temperature range (∆Tr), and gelatinization enthalpy (∆H) values for the various groups. The results indicate that the To, Tp, and Tc of starches obtained from HPP-treated pigeon pea milk increased significantly, whereas Tr and ∆H decreased significantly in the HPP-200 and HPP-400 groups. It could be observed that the endothermic peak shifted to the range of 50–80 °C and experienced a reduction in peak width and height with an increase in treatment pressure. When the pressure was increased to 600 MPa, the gelatinization peak disappeared and To, Tp, and Tc could not be measured, indicating the state of complete starch gelatinization. A similar DSC thermogram was obtained with the HTST group, which demonstrates that the increase of HTST treatment to a certain extent, produces a starch gelatinization-promoting effect similar to that of HPP treatment pressure. The lowest ∆H value was found in the HPP-200 group, whereas the lowest ∆Tr value occurred in the HPP-400 group due to increases in To, Tp, and Tc. This shows that starches obtained from samples subjected to HPP at 200 MPa maintained a better starch crystal structure compared with the HPP-400 samples. Among the various groups, the HPP-600 and HTST groups achieved 100% starch gelatinization, whereas the HPP-400 group exhibited the lowest degree of starch gelatinization.

### 2.4. In Vitro Digestibility Experiment

From Table 3, it can be seen that the resistant starch (RS), readily digestible starch (RDS), and slowly digestible starch (SDS) contents of untreated pigeon pea milk (control group) were 43.1%, 7.7%, and 15.3%, respectively. When HPP was performed at 200 MPa, the contents of the three types of starches increased significantly. In particular, the RS and SDS contents of the HPP-400 group reached 47.3% and 17.8%, respectively, which were the highest among all groups. For pigeon pea milk samples of the HPP-600 and HTST groups, the higher degree of starch gelatinization led to a significant decrease in RS and SDS content but an increase in RDS content, with the RS content of the HPP-600 group being the lowest among all groups. A similar trend was observed in the hydrolysis indices (HI) of the various groups. HI was significantly lower in the HPP-200 and HPP-400 groups, with the values being lower than that of the control group, while the HPP-600 group had the highest HI value, which was not significantly different from that of the HTST group. The glycemic indices (GI) of the control, HPP-200, HPP-400, HPP-600, and HTST groups were 50.4, 49.7, 49.1, 55.7, and 55.3, respectively. From these results, it can be deduced that HPP at 400 MPa effectively increased the RS and SDS content of pigeon pea milk and led to a reduction in GI compared to untreated pigeon pea milk. In contrast, excessive HPP promoted starch gelatinization, reduced RS and SDS content, and increased the digestibility and absorbability of starches in a similar manner to HTST pasteurization, which led to an increase in GI.

### 2.5. Scanning Electron Microscopy

**Figure 3:**
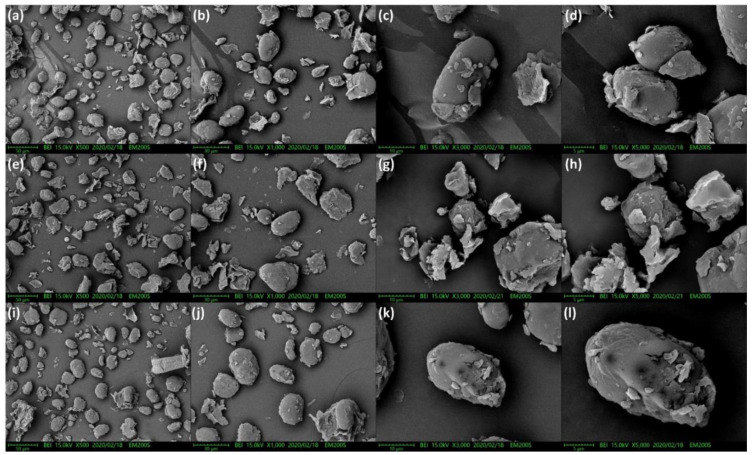
Scanning electron micrographs of starches from pigeon pea milk, (**a**–**d**) control; (**e**–**h**) HTST; and (**i**–**l**) HPP.

Figure 3 shows the SEM of the starch granules obtained from untreated pigeon pea milk and pigeon pea milk samples subjected to either HPP (400 MPa/210 s) or HTST pasteurization. Figure 3a–d, illustrate the scanning electron micrographs of the control group, it can be seen that the ungelatinized starch mainly existed as plump granules having diameters of 10–30 μm, with a small amount appearing as granule fragments. Figure 3e–h represent the scanning electron micrographs of the HTST group. It shows that high temperature increased the degree of starch gelatinization. Consequently, a large number of fragmented, irregularly-shaped starch granules as shown in Figure 3h, are markedly different from the intact starch granules exhibited in Figure 3d. Figure 3i–l show the starch granule morphology from HPP-treated pigeon pea milk. At 500× and 1000× magnifications, it can be seen that starch mainly existed as intact, plump granules with a small number of fragments. The scanning electron micrographs at 3000× and 5000× magnifications further indicate that HPP at 400 MPa did not result in a higher degree of starch gelatinization. Consequently, the starch granule morphology shown in Figure 3l was similar to that in Figure 3d (control group).

## 3. Discussion

In recent years, HPP has been applied to the pasteurization of a wide variety of legume-based beverages due to its ability to prolong shelf-life and retain nutritional attributes of food products. In a study by Smith et al. [11], it was found that pressures at 400 MPa (5 min), 500 and 600 MPa (1 and 5 min) produced 100% microorganism sub-lethal injury in soymilk. Fresh soymilk deteriorates after 7 days in refrigerated storage, and with HPP, the shelf-life of soymilk can be extended to at least 2 weeks. It has been found that a combination of high temperature (75 °C) and HPP (600 MPa/1 min) not only significantly reduced the initial microbial count and prolonged shelf-life, but also increased the stability of soymilk proteins. Andrés et al. [12] compared the effects of HPP and thermal pasteurization on the quality of soy-smoothies, and found that during a 45-day refrigerated storage experiment, the microbial safety achieved with HPP at 650 MPa for 3 min at 20 °C was comparable to that achieved with thermal pasteurization at 80 °C for 3 min. The contents of soluble sugars (glucose and fructose), organic acids (citric, malic, tartaric, oxalic, and quinic), and minerals (sodium, potassium, calcium, magnesium, iron, copper, zinc, and manganese) did not differ significantly between the HPP-treated and heat-pasteurized samples during the storage period, and no significant differences were found in sensory attributes between the untreated and HPP-treated soy smoothies. In another study, it was observed that with a combination of high pressure and mild thermal treatment (75 °C), HPP altered the isoflavone profiles of soymilk through promoting a shift in the isoflavone profile from malonylglucosides toward β-glucosides [17]. Tsai et al. [18] investigated the effects of HPP on the quality attributes of hazelnut milk during refrigerated storage, and found that HPP at 600 MPa for 5 min achieved the same level of microbial safety as thermal processing at 80 °C for 3 min and significantly decreased immunoreactivity without significantly changing the physicochemical properties of the hazelnut milk. In the present study, a pasteurization experiment was performed with HPP conditions that enabled a 5-log reduction of *E. coli* O157:H7, namely 200 MPa/240 s, 400 MPa/210 s, and 600 MPa/150 s. Results indicated that the inhibition of TAB, psychrotrophs, and *E. coli*/coliforms in HPP-treated samples were not significantly different compared to the HTST-treated samples up to 21 days of refrigerated storage. Although growth of the residual bacteria was detected on Day 28, the bacterial counts did not exceed 1 log CFU/mL. Therefore, the recommended shelf-life of HPP-treated pigeon pea milk should not exceed 21 days to maintain the minimum microbial safety.

Besides maintaining microbial safety in legume-based beverages, HPP can also alter gelatinization characteristics of starches and increase resistant starch content in these beverages. In a study by Li et al. [19], red adzuki bean starches were subjected to high HPP, and it was found that a pressure of 600 MPa induced a high degree of starch gelatinization. Consequently, DSC analysis revealed a significant decrease in gelatinization enthalpy, and a Maltese cross pattern was observed among the starch granules. Through electron microscopy, it was also found that starches subjected to pressures below 450 MPa mainly existed as oval-shaped, plump granules. When the starch samples were subjected to a pressure of 600 MPa, the granules appeared fragmented, irregularly-shaped, and had obvious creases due to starch gelatinization. Similar results were reported by Liu et al. [20], who investigated the properties of HPP-treated tartary buckwheat starch. Under an electron microscope, it was found that intact granular morphology could be maintained in starch samples subjected to HPP at lower pressures (120–480 MPa). However, when the treatment pressure was increased beyond 480 MPa, starch gelatinization occurred and led to the formation of lumps through the stacking of starch granules. In the present study, our results showed that HPP at 400 MPa did not cause excessive starch gelatinization and produced an increase in the resistant starch content of pigeon pea milk. Consistent findings were also obtained from SEM analysis, with the scanning electron micrographs showing intact starch granules similar to those observed with the control group. With HTST pasteurization, complete starch gelatinization occurred, which resulted in the fragmentation of starch granules in pigeon pea milk. These results indicate that HPP caused instability in the crystal sheet structures of starch and decreased the crystallinity of starch granules; thereby reducing the energy required for gelatinization and leading to a decrease in starch gelatinization temperature. As ∆Tr represents the stability of crystalline regions in starch granules, it is positively correlated with crystallinity and the strength of heterogenous crystals in starch. Changes in ∆H are associated with the destruction of the double-helix structures in starch. HPP promotes a decrease in ∆H through the gelatinization of certain straight-chain starch molecules, which may be caused by the destruction of crystalline regions and disintegration of the double-helix structures [21]. The ∆Tr values in Table 2 indicate that HPP significantly destroyed the crystal structures of starch, thereby reducing the stability of crystal regions within starch granules. ∆Tr could not be measured when the pressure was increased to 600 MPa, indicating the complete disintegration and gelatinization of starch crystals.

The results of the present study show that HPP treatment at 400 MPa resulted in RS and SDS contents of 47.3% and 17.8%, respectively, as well as a decrease in GI, which demonstrate the potential of pigeon pea milk as a health-promoting beverage for the regulation of blood glucose concentrations. Letite et al. [6] analyzed the influence of HPP at 300–600 MPa on the gelatinization characteristics of pea starch, and observed that curves obtained with samples subjected to HPP at 300–400 MPa exhibited a ∆H peak. In contrast, the curves of samples subjected to HPP at 500 or 600 MPa did not show any peaks, indicating that pressures of 500 MPa induced complete gelatinization in starch, whereas a pressure of 400 MPa only resulted in 31% gelatinization. Similar results were also obtained in the present study, in that at a treatment pressure of 600 MPa, a ∆H peak could no longer be seen in the DSC thermogram, and the degree of gelatinization reached 100%. When HPP treatment was performed at 400 MPa, To, Tp, and Tc were increased with only 18.4% of gelatinization occurred. Based on degradation time in the human intestine, starches can be classified as rapidly digestible starch (RDS), slowly digestible starch (SDS), and resistant starch (RS). Narina et al. [22] found that pigeon pea had a higher RS content compared to other food legumes, it was shown that chickpea and corn starch had 9.38% and 37.74%, while pigeon pea had 70.4%. However, the RS content of pigeon pea is influenced by cultivar type and processing conditions. Kasote et al. [23] analyzed the effect of HPP on the RS content and in vitro starch digestibility of pigeon pea, and found that HPP resulted in the conversion of RS and RDS to SDS, and significantly enhanced the GI of pigeon pea. These results are largely consistent with the findings of the present study, which indicate that HTST and HPP at 600 MPa increased GI value and reduced RS content without increasing SDS content. Therefore, HPP enables the reduction of RDS content, the increase of SDS and RS content, and decrease in the degree of hydrolysis of pigeon pea starches, thereby enhancing the potential health benefits of pigeon pea milk.

## 4. Materials and Methods

### 4.1. Pigeon Pea Milk Preparation

Pigeon peas were collected form Xinyi Township, Nantou, Taiwan in January, 2019. The beans were washed with running tap water for the removal of surface dirt and then soaked in water at room temperature for five hours. Subsequently, the water was drained and fresh water was added to the soaked pigeon peas (pigeon peas: water = 1:5) in an electric mixer and ground on low speed for one minute. Filtered pigeon pea milk was collected through pressing the resultant slurry in a 100-mesh nylon filter bag. The pigeon pea milk contains 80% of water and appearance milky white, pH is 6.6. The fresh pigeon pea milk was immediately subjected to pasteurization.

### 4.2. HPP

*E. coli* O157:H7 strain (BCRC No. 14824) was obtained from the Bioresource Collection and Research Center (BCRC) of Taiwan. The strain was cultured in nutrient broth containing 0.5% NaCl at 37 °C until the stationary growth phase had been reached. Bacterial cells were collected by centrifugation at 3000× g for 10 min and washed in phosphate buffered saline (PBS: 0.01 M phosphate, 0.137 M NaCl, pH 7.3). The washed bacterial cells were resuspended in pigeon pea milk at a concentration of approximately 1 × 10^8^ CFU/mL and sealed in a sterile vacuum package using a vacuum sealer. All samples subjected to HPP contained freshly prepared bacterial cultures obtained using the same procedure. The sealed pigeon pea milk samples were then loaded into the HPP equipment with a total vessel volume of 6.2 L and a maximum operating pressure of 600 MPa. Pressurization was performed using pure water as the pressure-transmitting medium, with the rate of pressurization being 300 MPa/min, and temperature increased at approximately 3 °C for every 100 MPa of increased pressure. After HPP treatment was completed, depressurization was performed over a duration of 5 s. Pigeon pea samples were treated at pressures of 400, 500, and 600 MPa for 30, 60, 90, 120, 150, 180, 210, and 240 s, respectively, at 10 °C. The log CFU/mL reductions for the different pasteurization conditions were calculated to determine the minimum threshold conditions required to achieve a 5-log reduction of *E. coli* O157:H7.

### 4.3. HPP and HTST Pasteurization

In accordance with the operating specifications for HPP recommended by the United States Department of Agriculture (USDA), HPP conditions that enabled a 5-log reduction of *E. coli* O157:H7 in pigeon pea milk were selected for the pasteurization experiment. Freshly prepared pigeon pea milk samples were inoculated with 1 × 10^5^ *E. coli* O157:H7 cells, sealed in sterile polyethylene bags and subjected to different HPP treatments (200 MPa/240 s, 400 MPa/210 s, and 600 MPa/150 s; at 10 °C) to form the HPP-200, HPP-400 and HPP-600 samples. Fifty milliliters of pigeon pea milk was subjected to thermal pasteurization at 90 °C for 1 min to form the HTST sample group. After pasteurization, the pigeon pea milk samples were kept in refrigerated storage at 4 °C for a 28-day storage experiment. During the storage period, sampling of the unopened HPP-treated pigeon pea milk sample at each time was performed once every 7 days, and the samples were compared to the control (untreated sample) and HTST sample groups. The total aerobic bacteria (TAB) count, psychrotrophic count, and *E. coli*/coliform count in pigeon pea milk were determined using 3M Petrifilm^TM^ count plates (3M Corp., St. Paul, MN, USA). Pigeon pea milk samples were serially diluted using sterile 0.1% buffered peptone water and inoculated onto the count plates. The count plates were incubated under the following conditions: TAB count: 30 °C for 24 h; psychrotrophic count: 10 °C for 5 days; and *E. coli*/coliform count: 37 °C for 48 h. After culturing, count plates with 25–250 bacterial colonies were selected for the recording of colony counts, with 1 CFU/mL as the minimum detection limit.

### 4.4. Degree of Gelatinization

The gelatinization studies were carried out according to method described by Leite et al. [24] with some modifications. Pigeon pea milk samples were subjected to HPP and HTST pasteurization and centrifuged at 1500× g for 10 min. After centrifugation, the supernatant was removed and the precipitate was air-dried at 30 °C for 24 h, ground, and passed through a 100-mesh sieve to facilitate analysis using a differential scanning calorimeter (Setline^®^ DSC, Setaram Instrumentation, Caluire-et-Cuire, France). Starch gelatinization in the samples was examined using a modified version of the method reported by Wada et al. [25]. Approximately 5–6 mg of sieved pigeon pea starch powder was obtained, and water as much as four times the mass of the starch powder was added to achieve a final water content of 80%. The mixture was then placed in an aluminum crucible, sealed, set aside for 30 min, and subjected to DSC analysis with a heating rate of 5 ℃/min and temperature increased from 25 °C to 130 °C. For each sample, the onset temperature (To), peak temperature (Tp), and conclusion temperature (Tc) were determined from the DSC thermogram and the enthalpy of gelatinization (ΔH) was calculated.

### 4.5. In Vitro Digestibility Experiment

In vitro starch digestibility was analyzed using the method reported by Englyst et al. [26]. One milliliter of amyloglucosidase was added to 2 mL of deionized water. Next, 3.89 g of porcine pancreatic alpha-amylase was dissolved in 25.7 mL of water and centrifuged at 2500× g for 10 min, and 18.7 mL of the supernatant was collected. The supernatant was mixed with 1 mL of the amyloglucosidase solution for the preparation of an enzyme solution, with a fresh solution prepared prior to each digestion analysis experiment. Aliquots of guar gum (10 mL, 5 g/L) and sodium acetate (5 mL, 0.5 M) were added to test tubes containing 0.5 g of the starch sample. Seven glass balls (10 mm in diameter) and 5 mL of the enzyme solution were added to each test tube, and shaken at 170 rpm in a 37 °C water bath. Aliquots of 0.5 mL were removed at fixed intervals and mixed with 4 mL of 80% ethanol, and the glucose assay kits was used to determine glucose contents in the mixtures. The total starch content were classified based on digestibility as follows: readily digestible starch (RDS) for starch that was hydrolyzed within 20 min; resistant starch (RS) for starch that was not hydrolyzed within 120 min; and slowly digestible starch (SDS) for starch that was digested during the period between 20 and 120 min. Using the hydrolysis curve (0–180 min), the hydrolysis index (HI) was calculated as the percentage of total glucose released from the starch sample compared to that released from the white bread. The glycemic indices (GI) of the samples were estimated according to the equation proposed by Goñi et al. [27]: GI = 39.71 + 0.549HI.

### 4.6. Electron Microscopy

The starch granules in pigeon pea milk were observed by scanning electron microscopy (SEM). Starch samples were sputter-coated with platinum (TEMIC Mini Coater Alice-1000) and observed under a scanning electron microscope (TEMIC EM200S, Tainan, Taiwan). SEM images were captured at an accelerating voltage of 15 kV and magnification levels from 500–5000 times.

### 4.7. Statistical Analysis

All experiments in the present study were performed in triplicate. Experimental data were statistically analyzed using the Statistical Analysis System (SAS) software (STATISTICA^®^, 1999 edition, StatSoft Co., Tulsa, OK, USA). Data were analyzed by one-way ANOVA followed by Duncan’s multiple range test for the detection of inter-group differences. A significance level of α = 0.05 was used.

## 5. Conclusions

In the present study, the effects of HPP and HTST pasteurization on the microbial shelf-life, starch gelatinization characteristics and RS content of pigeon pea milk were investigated. Results indicate that HPP at the range of 200–600 MPa with appropriate pasteurization duration could achieve a 5-log reduction of the indicator bacteria, which meets the microbial safety requirements for cold chain courier. Through DSC and SEM analysis, it was found that excessive HPP at 600 MPa and HTST were unsuitable for the pasteurization of pigeon pea milk as these treatments induced a high degree of starch gelatinization and reduced the RS content. In contrast, HPP at 400 MPa required a longer pasteurization time but with a low degree of starch gelatinization while significantly increased RS and SDS contents, and reduced the GI of pigeon pea milk. These results are useful to understand the phenomenon of gelatinization induced by different conditions of HPP, which forces the entrance of water inside the pigeon pea starch granule, inducing the cold gelatinization. This study demonstrate that appropriate HPP conditions enable the extension of the shelf-life and enhancement of nutritional value of pigeon pea milk, thereby providing great potential for the application of pigeon pea milk in the development of health-promoting beverages for the regulation of blood glucose levels.

## Figures and Tables

**Table 1:** Bacterial count in control and pasteurized pigeon pea milk during storage at 4 °C.

	**Treatment**	**Time (Days)**					
		0^†^	0^‡^	7	14	21	28
TAB (log CFU/mL)	Control	1.65 ± 0.23Ad	-	2.82 ± 0.34Ac	4.12 ± 0.27Ab	5.57 ± 0.34Aa	5.42 ± 0.28Aa
	HPP-200	1.48 ± 0.28Aa	ND	0.31 ± 0.08Be	0.59 ± 0.11Bd	0.71 ± 0.14Bc	1.12 ± 0.21Bb
	HPP-400	1.72 ± 0.19Aa	ND	ND	0.27 ± 0.09Bd	0.46 ± 0.10Bc	0.78 ± 0.15Bb
	HPP-600	1.55 ± 0.14Aa	ND	0.23 ± 0.09Be	0.44 ± 0.17Bd	0.69 ± 0.13Bc	0.92 ± 0.12Bb
	HTST	1.51 ± 0.16Aa	ND	ND	ND	0.41 ± 0.15Bc	0.63 ± 0.11Bb
Psychrotrophs (log CFU/mL)	Control	1.34 ± 0.24Ae	-	2.15 ± 0.21Ad	2.33 ± 0.17Ac	3.21 ± 0.14Ab	3.82 ± 0.37Aa
	HPP-200	1.60 ± 0.27Aa	ND	ND	0.38 ± 0.10Bc	0.59 ± 0.11Bb	0.66 ± 0.17Bb
	HPP-400	1.46 ± 0.21Aa	ND	ND	0.22 ± 0.14Bc	0.64 ± 0.11Bb	0.77 ± 0.09Bb
	HPP-600	1.59 ± 0.17Aa	ND	ND	0.30 ± 0.08Bd	0.77 ± 0.17Bc	0.92 ± 0.18Bb
	HTST	1.52 ± 0.29Aa	ND	ND	ND	ND	0.72 ± 0.11Bb
*E. coli*/Coliforms (log CFU/mL)	Control	0.35 ± 0.16Ae	-	0.51 ± 0.29Ad	0.47 ± 0.12Ac	0.89 ± 0.15Ab	1.77 ± 0.24Aa
	HPP-200	0.47 ± 0.17Aa	ND	ND	ND	0.33 ± 0.07Bb	0.65 ± 0.11Ba
	HPP-400	0.39 ± 0.09Aa	ND	ND	ND	ND	0.48 ± 0.13Ba
	HPP-600	0.45 ± 0.11Aa	ND	ND	ND	ND	0.57 ± 0.08Ba
	HTST	0.38 ± 0.12Aa	ND	ND	ND	ND	ND

All data were the means ± SD (*n* = 3). 0^†^, day 0 results before treatment; 0^‡^, day 0 results after high pressure processing (HPP) or high-temperature short-time (HTST) treatment. TAB—total aerobic bacteria; -—not analyzed; ND—not detectable (<1 CFU/mL). Different capital letters in the same column indicate significant statistical differences; different lowercase letters in the same row indicate significant statistical differences (*p* < 0.05).

**Table 2:** Values of thermal properties by DSC analysis of pea starch processed by HPP in pigeon pea milk.

Treatments	To (°C)	Tp (°C)	Tc (°C)	∆Tr (°C)	∆H (J/g)	% G
Control	39.54 ± 1.21b	70.09 ± 1.50b	80.20 ± 1.31b	41.65	645.53 ± 46.64a	0
HPP-200	51.83 ± 1.63a	70.81 ± 0.25b	84.52 ± 0.38a	31.18	490.84 ± 25.38b	23.9
HPP-400	54.34 ± 1.34a	75.70 ± 1.73a	84.77 ± 0.61a	30.59	526.73 ± 53.18b	18.4
HPP-600	ND	ND	ND	ND	ND	100
HTST	ND	ND	ND	ND	ND	100

All values are means of triplicate determinations ± SD (*n* = 3). Means within columns with different letters are significantly different (*p* < 0.05). To—onset temperature; Tp—peak temperature; Tc—conclusion temperature; ∆Tr—gelatinization temperature range (∆Tr = Tc−To); ∆H—enthalpy of gelatinization; ND—not detected.

**Table 3:** Digestibility properties of starches from different pigeon pea milk.

Treatment	RS (%)	RDS (%)	SDS (%)	HI	GI
Control	43.1 ± 0.4c	7.7 ± 0.7d	15.3 ± 0.3b	19.5 ± 0.4b	50.4 ± 0.4b
HPP-200	44.1 ± 0.5b	11.5 ± 0.1c	17.6 ± 0.5a	18.2 ± 0.4c	49.7 ± 0.3ab
HPP-400	47.3 ± 0.6a	11.1 ± 0.2c	17.8 ± 0.6a	17.1 ± 0.5d	49.1 ± 0.4c
HPP-600	17.5 ± 0.1e	20.2 ± 1.3a	9.2 ± 0.4d	29.1 ± 0.6a	55.7 ± 0.5a
HTST	22.7 ± 0.1d	19.8 ± 0.6b	11.5 ± 0.4c	28.4 ± 0.4a	55.3 ± 0.3a

All values are means of triplicate determinations ± SD. Means within columns with different letters are significantly different (*p* < 0.05). RDS—readily digestible starch, SDS—slowly digestible starch, RS—resistant starch, HI—hydrolysis index, GI—glycemic index.
